# How Do We Get the Public Into Public Health Research? Learnings and Key Recommendations From Initiating a Community Involvement Project Scheme

**DOI:** 10.1111/hex.70114

**Published:** 2024-12-08

**Authors:** Carmel McGrath, Gemma Lasseter, Noreen Hopewell‐ Kelly, Emma Anderson, Ellen Brooks‐Pollock, Hannah Christensen, Sarah Denford, Rosie Essery, Shoba Dawson, Evelyn Schiller, Taru Silvonen, Christina Stokes, Amy Thomas, Clare Thomas, Andy Gibson

**Affiliations:** ^1^ NIHR Health Protection Research Unit in Behavioural Science and Evaluation, Population Health Sciences, Bristol Medical School University of Bristol Bristol UK; ^2^ The National Institute for Health and Care Research Applied Research Collaboration West University Hospitals Bristol and Weston NHS Foundation Trust Bristol UK; ^3^ Faculty of Health and Applied Sciences, School of Health and Social Wellbeing University of West England Bristol UK; ^4^ Population Health Sciences, Bristol Medical School University of Bristol Bristol UK; ^5^ Division of Population Medicine, Marie Curie Palliative Care Research Centre Cardiff University Cardiff UK; ^6^ Centre for Academic Child Health, Population Health Science, Bristol Medical School University of Bristol Bristol UK; ^7^ Primary Care Research Centre, School of Primary Care, Population Science and Medical Education University of Southampton Southampton UK; ^8^ Sheffield Centre for Health and Related Research University of Sheffield Sheffield UK

**Keywords:** community involvement, learning, PPI, public health research, public involvement, reciprocity, UK Standards for Public Involvement

## Abstract

**Introduction:**

There are many recognised benefits of public involvement, including more relevant research. The COVID‐19 pandemic highlighted the existing health inequalities and disparities in access to care and treatment for under‐served groups, necessitating meaningful and sustainable approaches to engaging them in health research. However, there is limited guidance to suggest what groundwork and processes are necessary for initiating such projects. This paper outlines the practical approaches taken to initiate a community involvement project scheme and offers key recommendations from this work.

**Methods:**

The National Institute for Health and Care Research (NIHR) Health Protection Research Unit established a community involvement scheme in 2021, funding four community involvement projects enabling researchers to engage with under‐served communities. Reflections were captured through regular quarterly meeting group discussions, meeting notes and email correspondence.

**Results:**

The paper presents the steps taken to initiate a scheme that provided funding for a diverse range of projects working with under‐served communities. The projects demonstrated the value of allocating time to build relationships and trust, maintaining flexibility, and providing short‐term benefits such as remuneration and training to the community.

**Discussion:**

This paper has highlighted the need for research organisations to allocate funding and resources within their infrastructures for building trusting relationships with community leaders and communities.

**Conclusion:**

This paper has outlined the steps undertaken to engage with under‐served communities to bridge the gap between public health research and those communities. We present key recommendations to guide future initiatives aspiring to engage under‐served communities in health research.

**Patient or Public Contribution:**

Public contributors have been involved in all of the four community involvement projects mentioned in this paper. Two public contributors are also co‐authors and have provided input to the writing and review of this manuscript.

## 1 Introduction

For democratic, political, and practical reasons, involving patients, the public, and service users as partners in research is increasingly recognised as important by policymakers, funders, and those undertaking research [1, 2, 3]. There are many recognised benefits of public involvement including research that is considered more relevant, equitable, and sustainable [3, 4, 5, 6]. However, the COVID‐19 pandemic further accentuated the existing health inequalities and disparities in access to care and treatment for under‐served groups [7, 8, 9, 10].

It is important to acknowledge that there are different factors including, political, economic, and cultural issues, that may influence which research is conducted, who conducts it, and whether the findings are implemented [11]. Importantly, there can be a significant gap between the priorities and values that underpin the systems that govern research and the needs and concerns that emerge from the lifeworld of people in underserved communities. Public involvement is a key process that can close the gap between the ‘system’ and the everyday lives of individuals, but whether it is successful in this depends on whether it is designed and carried out in a way that is sensitive to the individual needs of under‐served communities [12].

It has been acknowledged that there is no single definition that can be used to describe all under‐served groups. The National Institute for Health and Care Research (NIHR) recently established key characteristics that are common to under‐served groups [13]. These characteristics include lower inclusion in research when compared to population estimates, higher healthcare burden that is not addressed by research, and a lack of recognition and response to how different groups engage with healthcare interventions [13]. Examples of under‐served groups include those who are unemployed or have low incomes, people who have learning disabilities, people who have language barriers, and people from minority ethnic groups [13]. The lack of inclusion of these groups limits the generalisability, relevance and accessibility of the research and interventions produced. As a result, such interventions may not meet the needs of the wider population further exacerbating healthcare inequalities [13]. Developing meaningful and sustainable approaches to working with under‐served groups that do not often engage in research is, therefore, crucial to begin addressing these key health issues [14, 15].

Existing literature and guidance provide information and recommendations on working with under‐served communities in different areas of research, including global health [10, 13, 16, 17, 18, 19]. For example, the Health Innovation East Midlands have produced short guides to support public involvement for example, a guide on how to co‐produce with the Adult Learning Disability Community and top tips for engaging with Asylum Seekers and Refugees [20]. The guidance highlights the need to invest in building trust and relationships due to the previous stigma, discrimination, and exclusion experienced by under‐served communities.

The NIHR have recently published guidance on ‘Being inclusive in public involvement and health and care research’ [21]. This guidance has been produced to support researchers in undertaking public involvement and is based on work by Prof. Starling and the experiences of the NIHR INVOLVE Diversity and Inclusion Group. The guidance prompts researchers to consider the differences in power between people, researchers, and institutions, commit to relationship building, and invest in supporting people to develop confidence and learn new skills [21].

Masood and colleagues have published a series of papers detailing strategies to improve the inclusivity of minoritised communities in health research [22]. In one paper, aimed at assisting General Practitioners (GPs) and primary care researchers in developing inclusive public involvement practices, they restate the importance of overcoming structural and cultural barriers, addressing socioeconomic challenges, and building trust with minoritised communities [22].

As highlighted by these examples, working with under‐served communities involves investing time, and resources, relationship building and breaking down structural barriers to plan research with the community and establish partnerships [16, 19]. However, there is relatively little practical guidance that suggests what approaches can be used to set up an infrastructure within an organisation aiming to bridge the gap between research and under‐served communities.

The purpose of this paper is to provide an account of the practical and accessible strategies employed in four community involvement projects that took place to initiate the involvement of under‐served communities within the context of public health research (see Table 1). We provide key recommendations based on our reflections which will be helpful for others who aspire to set up community involvement initiatives (Table 4).

**TABLE 1:** Brief overview of the four community involvement projects.

Community project	Information about the community project	Original project start and end dates and funding amount awarded
Project one ‘Hold the door open’	Involving older adults from diverse backgrounds in health research. The project aims to develop meaningful ways to share research findings together with people aged 55 + . For various reasons, such as limitations in access to information, health concerns and lack of access, older adults may be likely to be involved and represented in health research [23, 24]. However, with an increasingly ageing population, it is important that older adults are able to participate in research [13, 25]. Furthermore, there is a recognised need for the inclusion of voices from less affluent individuals and minority ethnic groups who are currently underrepresented in public involvement. Work has been ongoing since August 2021 to co‐design and co‐deliver events with public contributors to find new ways to work with and involve older adults in research [26].	August 2021– March 2023 (8 months) £6308
Project two ‘Facilitating the inclusion of voices of underrepresented groups in the work of the HPRU’	The COVID‐19 pandemic has disproportionately impacted certain populations, including people belonging to minority ethnic groups and those in more deprived regions and areas. Young people from these groups are already underrepresented in research and health service provision, and in an emergency situation, their voices risk exclusion altogether. This project aims to find better ways of communicating and working with young people from under‐served groups and communities and conducting research in a more collaborative way.	June 2021–Spring 2024 (32 months) £1000
Project three ‘Engaging the farming community in zoonotic disease research’	The farming community is a unique cohort of individuals who are often underrepresented in community involvement panels. Their occupation does not lend itself easily to involvement opportunities due to long, inflexible working hours and living in isolated, rural communities. This community project embeds farming representatives into zoonotic disease research.	June 2020–June 2021 (12 months) £2712
Project four ‘Maternal Vaccination in the NHS (MAVIS) Study – engagement with under‐served communities’.	The MAVIS Study aims to understand why huge disparities exist across regions and demographic groups (especially ethnicity) in the uptake of maternal vaccinations, and what can be done about it. Mothers with low vaccine confidence and those from under‐served communities need to be represented in this research if is to achieve its intended impact on addressing inequity. This community project aimed to engage an advisory group of mothers from Black and other minority ethnicities and ideally those who were unconfident about vaccination to coproduce research design and delivery, to explore reasons for low vaccine uptake and to inform intervention/policy recommendations.	June 2021–June 2024 (36 months) £9980.

## 2 Methods—Brief Project Background and Reflection Process

The NIHR Health Protection Research Unit in Behavioural Science and Evaluation (HPRU BSE) is a collaboration between the UK Health Security Agency (UKHSA) and partnership with Universities [27]. The HPRU where these projects are based is one of 14 virtual HPRUs working across England on research topics in predetermined themes including antimicrobial resistance and infectious diseases [28]. The research conducted by the HPRUs supports the UKHSA in protecting the public's health and minimising the health impact of emergencies [27].

In 2021, the HPRU BSE created a Community Involvement Project (CIP) scheme that enabled affiliated researchers to apply for funding, from a pot of £20K, to engage with under‐served communities [29]. In the wider literature, it has been acknowledged that building relationships of trust and reciprocity with communities is a process that requires time and resource [16]. Practical issues such as working on research projects with limited funding and timescales can lead to short‐term and extractive interactions resulting in frustration and disengagement from communities [16]. It was anticipated that this funding would equip researchers with the resources necessary to establish reciprocal relationships with under‐served communities and provide opportunities for communities to shape existing and potential future research projects [29]. The CIP proposals were reviewed and judged by the HPRU BSE Programme Manager, two Public Involvement Leads, and public contributors who are members of the HPRU BSE Public Involvement Strategy Group. This resulted in the funding of four CIPs with each research team receiving varying amounts of funding [29, 30].

The steps taken to initiate the CIPs within their first year of running have been aligned to the UK Standards for Public Involvement (see Table 2) [31]. The standards provide guidance for reflecting on and improving the purpose, quality, and consistency of Patient and Public Involvement (PPI) practices and are designed for people and organisations that conduct research, support research, and promote PPI to improve research [31]. In this paper, the standards have provided a useful benchmark, enabling us to demonstrate how our practices were consistent with the current guidance.

**TABLE 2:** The UK Standards for Public Involvement including the accompanying descriptors [22].

UK Standards for Public Involvement	Descriptor
Governance	Involve the public in research management, regulation, leadership and decision making.
Support and Learning	Offer and promote support and learning that builds confidence and skills for public involvement in research.
Working Together	Work together in a way that values all contributions, and that builds and sustains mutually respectful and productive relationships.
Inclusive opportunities	Offer public involvement opportunities that are accessible and that reach people and groups according to research needs.
Communications	Use plain language for well‐timed and relevant communications, as part of involvement plans and activities.
Impact	Seek improvement by identifying and sharing the difference that public involvement makes to research.

The reflections reported in this paper were captured through regular quarterly meeting group discussions, meeting notes, and email correspondence. Subsequent meetings were held with the authorship team to discuss the structure and content of the paper.

## 3 Results

### 3.1 Aligning Practices Used to Initiate the Community Involvement Projects to the UK Standards for Public Involvement

**FIGURE 1:**
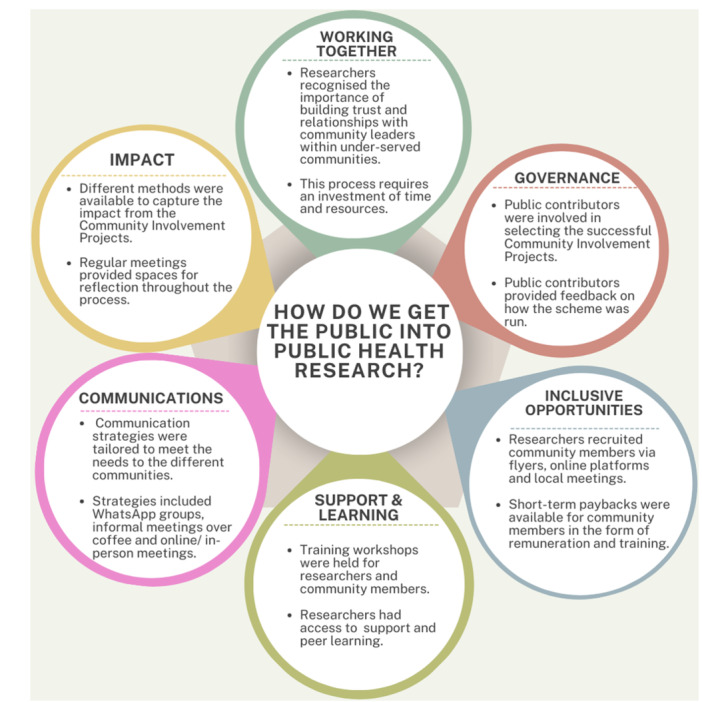
A graphic outlining the activities used to initiate the CIPs aligned to the UK Standards for Public Involvement in research.

This section of the paper details how the early activities used to initiate the CIPs aligned with the UK Standards for Public Involvement in research. The illustration presented in Figure 1 provides an outline of these steps.

#### 3.1.1 Governance‐ Reviewing the CIP Proposals, Selecting the Projects, and Influencing Decisions About the Aims of the Projects

All HPRU BSE Public Involvement Strategy Group members were invited to attend a discussion about the proposal for the scheme and were subsequently invited to volunteer to join the review panel. Two strategy group members put themselves forward for this opportunity and were involved in the process of selecting which community projects were awarded funding with the support of the public involvement team.

Ahead of the prioritisation meeting public contributors were asked to assess each project against three criteria: (i) aims and objectives, (ii) deliverability, and (iii) impact. Public contributors were provided with a template to fill in for each project and asked to provide a score from 1 to 10 for each criteria. At the meetings itself, deliberations began with a general discussion of the strengths of each project. This allowed people to develop an initial assessment. This initial assessment was then adjusted upwards or downwards based on more specific feedback about the precise details of each project. In this way consensus was achieved through discussion and a final score allocated to each project.

Involving public contributors in this way meant that the public voice was present in strategic‐level funding decisions and that the projects selected were considered important to the public involvement community. There were opportunities for reciprocal learning; public contributors experienced the process of reviewing grant applications and could gain a better understanding of the proposed projects.

The public involvement team developed mechanisms for supporting public contributors to be effectively involved in these decisions. The public contributors were offered opportunities to meet with the public involvement team before starting the process to make sure they had all the information they needed, to provide opportunities for the public contributors to ask questions, and to also provide any feedback on the selection process. After the process had been completed, the PPI team and public contributors reflected on the experience to identify the successes, challenges, and areas for improvement.

The involvement of public contributors at this stage helped ensure that a wider range of perspectives were included in the deliberations and a varied range of under‐served communities were represented in the projects. For example, the inclusion of a project focusing on the farming community was strongly supported by our public contributors as they recognised there are a range of communities who under‐served by research and the projects funded needed to reflect this.

One of the public contributors involved in the process and co‐author of this paper has reflected on their involvement in the selection of CIP projects. From their perspective, they felt very well supported by the PPI co‐ordinators. The public contributors were listened to, and their opinions were given equal weight in the discussions. The process helped the public contributors have a good understanding of the projects and they also felt it benefitted the selection process to have a good understanding of what was important to the public. The in‐depth knowledge that some of the PPI contributors had of under‐served communities added depth to the discussions. In summary, it was felt that this was a very positive experience for everyone involved.

#### 3.1.2 Support and Learning—What Opportunities for Support and Learning Were Offered?

Once the four projects had been selected, the researchers working on each CIP were provided with training in Public Involvement by experienced members of the HPRU BSE public involvement team and HPRU public contributors. The workshops included ‘An Introduction to Public Involvement’ and ‘Evaluating the impact of public involvement’ which were adapted from the workshops developed as part of the People in the Health West of England (PHWE) Learning and Development programme.

This training equipped the researchers who would be undertaking the CIPs with knowledge of the current guidance around involving people in research and included topics relating to payment processes and ideas for public involvement activities. During the evaluating impact session, the researchers were prompted to consider the approach that they would use to capture and evaluate the CIPs. The early training opportunity also created a space for the researchers to share ideas, contacts and collaboratively work through queries with colleagues and the public involvement team.

The researchers were also offered bespoke training and advice from the public involvement team that related to their projects. These sessions allowed researchers to consider and discuss initial plans for how they would begin their projects and implement evaluation within it. Regular four‐ monthly check‐in meetings were set‐up to ensure that the researchers received ongoing support and could have a forum to share and discuss any challenges and identify potential solutions together.

The funding offer for these projects was built on the principle of fostering reciprocal relationships and learning, as we recognised the crucial role it plays in building meaningful relationships with under‐served communities. By offering continuous opportunities for growth and development, we aimed to empower these communities and provide them with tangible benefits that would have a lasting impact.

The researcher working on the MAVIS project ensured that each face‐to‐face meeting with the community members included spaces for socialising. Additionally, training and learning opportunities were recurring items on the agenda.

To date, two training events have been organised for the community members involved in the MAVIS project on the topics of (i) communication skills and confidence and (ii) understanding research (e.g., the difference between qualitative and quantitative research and how to critically read news reports of health research). The communication skills teaching was delivered by an external training provider and paid for from the project budget. The training on understanding research evidence was delivered by an internal HPRU colleague.

The researcher working on the MAVIS project also linked the group with other research involvement opportunities, involved them in video production and is now exploring training so that they can conduct qualitative interviews or help to run focus groups, and co‐ present the study results.

The following section has been written by a community member who co‐facilitated the sessions as part of the MAVIS project. They reflect on their conversations with the community members and highlight the benefits of receiving support and opportunities for learning as part of their involvement.

*'The workshops presented a first opportunity for all participants to peek into the world of community research, breaking down the walls and simplifying the jargon that surrounds community research. As part of the sessions, the group explored the topic of communication and week by week, the confidence amongst participants grew exponentially. Most of all, involvement in this project brought voice, influence, and agency into the fabric of community, through our participation. It is about the ability for the community to have a voice and the skills and capabilities needed to realise their potential in both private and public spaces, both individually and through organising collectively. It is about the group members self‐confidence and self‐esteem, and ability to influence decisions and to make choices affecting people's lives. It is a learning curve that the group has only just stepped into but are all so proud about' (Community member and Co‐facilitator MAVIS study)*.

#### 3.1.3 Working Together—What Were Important Steps for Building Relationships and Working Together With Under‐Served Communities At the Early Stages?

Table 3 provides a detailed overview of the different approaches used by the researchers to engage with the communities. As shown in the table, the researchers dedicated time to focus on building relationships with community leaders, and/or community groups who had relevant experience and an interest in the community project. The relationship‐building process involved researchers meeting up for coffees in person or online with the community members to talk about the projects as well as attending pre‐existing community meetings or groups to introduce their research project in a relaxed and informal environment. For the MAVIS project, the community member was involved in recruiting people to the group. In the following paragraph, they provide further information about their experience of this process.

**TABLE 3:** A table outlining how each project identified and recruited community members.

Community project name & Researchers	Steps taken to identify and recruit community members
Project one ‘Hold the door open’	As the project was based in locations across the UK, the researchers lacked established relationships with local older adult community groups. As a first step, the researchers contacted community leaders and group administrators who had existing connections and knowledge of the appropriate channels to share information about the project with older adults. The researchers were also advised to advertise through community groups via social media.
Project two ‘Facilitating the inclusion of voices of underrepresented groups in the work of the HPRU’	The researchers did not have existing relationships with children and young people (CYP) or organisations working with CYP from under‐served communities. This proved challenging when trying to recruit CYP as attempts made via email and phone to connect with community organisations were largely unsuccessful. However, the researchers were invited to present at a network meeting that brought together local community organisations working with CYP. Through this presentation, a connection was made with an individual working at the Creative Youth Network. This individual played a critical role in facilitating contact with community organisations which resulted in successful recruitment and further workshops being held.
Project three ‘Engaging the farming community in zoonotic disease research’	Twitter was used as a platform to recruit farmers, as the researchers did not have pre‐existing relationships with the farming community and wanted to make sure they reached farmers from across the UK. From this advertisement, four farmers who were either active or retired were recruited to an advisory panel. As a result of the relationships built with the panel members, they then successfully recruited a public contributor from this panel who was involved in co‐developing the zooTB study [32, 33].
Project four ‘Maternal Vaccination in the NHS (MAVIS) Study—engagement with under‐served communities’.	Recruitment for this project involved making connections through the PPI Lead who had existing community contacts and could identify individuals who may be interested in working on the project. The researcher also worked with a Ugandan health advocate, a Bangladeshi mother, and community leader, who were recruited to identify and engage women from their communities and facilitate workshops. The community contacts were extensively involved in the planning and several face‐to‐face and Zoom meetings were held with them to discuss and plan the project. They also spent time building trust with mothers they knew to invite them to attend the workshops. However, two contacts were concerned about being exposed for their anti‐vaccination views due to the pandemic and the possibility of vaccine passports and restrictions on freedom and did not want to attend project meetings.

*'Introducing research to the ladies was not as challenging as first anticipated. The women who were approached to be involved, were keen to be part of the change they want to see in their communities. The MAVIS project team needed to put them, their families, and friends into the frame of the project and its aims, so that a shared understanding was quickly reached. The critical aspect of recruitment lay in supporting them to understand the power of collective action in community research. This relates to the power within each individual in the group, the power manifesting with the group as a whole and the power of the group in its role and topic of community engagement. A keen interest, connection, and eagerness to progress remained among the women, not to mention the significant interest expressed by other women in joining the group for community research' (Community member and Co‐facilitator MAVIS study)*.

Challenges arose when the researchers and public involvement team did not have established links with communities or community organisations. At the time of setting up the projects, the COVID‐19 pandemic was preventing in‐person meetings so initial contact to prospective community organisations and individuals was made via email and phone calls. This approach was not always effective, and emails and calls were often unanswered. Over time, it was possible for the researchers to work with community organisations, but this was facilitated only through connections with individuals who had pre‐existing links with the communities and/or community organisations.

The researchers working on the project ‘Hold the door open’ (Project One) relied on a combination of previous contacts and community organisations to share information about opportunities via email. Leads created through an ongoing UK‐wide research project facilitated contacting people across the UK. Major organisations such as Age NI and Age Scotland were particularly helpful in sharing information about opportunities for the activities aimed at people aged 55+ and taking place outside of the research setting. The researchers working on Project Two (see Table 3 for further information) did not have pre‐existing relationships with children and young people from under‐served communities or organisations working with this community. However, it was through an individual working at the Creative Youth Network that they were introduced to different organisations, which then enabled the recruitment of young people and subsequent workshops to be held.

In some cases, members of public involvement teams (external to the HPRU) had pre‐existing links with community leads. In these circumstances, the researchers were supported by these external public involvement teams to make the initial contact with communities. Where relationships did not exist, the HPRU public involvement team worked with the researchers to identify and explore possible contacts. These examples have illustrated the unseen time and resources necessary when working with communities and building these important partnerships.

#### 3.1.4 Inclusive Opportunities—How Were the CIPs Opportunities Offered to the Communities?

To make sure a range of people could be involved in the community projects from the earliest phase, the researchers invested time to consider which perspectives would be relevant to their projects and plan the approaches that they would use to work together with these community groups. The researchers detailed this information in their community project applications. This meant that once funding had been received, the researchers could adapt and tailor their approaches (with advice and support from the public involvement team) to recruiting community members from the outset.

Each project aimed to recruit people from different communities therefore a range of approaches were used to advertise opportunities to join the CIPs (see Table 3). Some researchers were given permission by community leaders to attend pre‐existing meetings, whilst other researchers posted and advertised through social media.

Where possible, the researchers met with community members in settings that were friendly, relaxed, and accessible. When meetings were in person, it was possible for some of the researchers to provide refreshments and payment for the community members and community lead's time and travel in the form of vouchers or bank transfer depending on the preference of the individuals. The payment rate was set in line with the current NIHR payment guidance [34]. Based on the feedback from community groups it was apparent that incentives such as food, vouchers and payment were well received and appreciated by the groups. Some groups expressed the need to provide incentives when working with communities. In the next paragraph, the community member who co‐facilitated the MAVIS sessions explains the strategies used to prevent potential barriers to involvement.

*'Research is considered a big scientific unreachable expert process by people in the community. It was important when inviting them to be involved in research to convey to the women how they could be involved in shaping the research process. To do this, when speaking to the women, the use of jargon was avoided, including the word 'research'. Instead, the team talked about 'community engagement', and the importance of the women sharing their “take and views” on certain issues affecting communities through 'workshops' and holding 'focus group discussions' and 'giving feedback'. Once the group members understood how they could be involved and make a difference throughout the research process there was no turning back. The group were keen to be involved, to stay put and march proudly onwards' (Community member and Co‐facilitator MAVIS study)*.

#### 3.1.5 Communications—What Were the Communication Strategies Used to Reach Out to Community Groups?

In recognition that the projects were aimed at reaching out to those under‐served communities who do not often get involved in research, it was crucial to ensure that communications strategies were tailored to meet the needs and preferences of the communities [19]. Across the four projects, the researchers used a blend of communication methods to reach out and recruit members for their projects (see Table 3). These approaches included emails, face‐to‐face and online meetings, sharing flyers, providing visual invitations that could be shared via WhatsApp, meeting up for coffee, sharing details of the projects on websites and attending pre‐existing groups. The public involvement team also supported the researchers to create materials to share information about their projects so that they were accessible to a range of audiences. As previously mentioned, as part of their communications plans, the researchers were encouraged to consider how they would evaluate their projects and provide feedback to the communities they were working with.

The community member and co‐facilitator working on the MAVIS project reported how they had initially started off planning to communicate with the group via email, however they realised that this was an impractical method of communication to ask mothers of children who were going to school and trying to juggle work, household, and family life. Instead, it was realised that the group members preferred “WhatsApp” as all members had a phone and could quickly read and respond to messages. Once the group got to meet and know each other, phone calls and group messaging were used by the group to interact. Through this ease of communication, the group has been able to engage, share and gain knowledge with each other which has, in turn, increased their self‐esteem and self‐belief. The group are more outgoing and outspoken than when the project started, and this frequent interaction has also resulted in new friendships forming within the group.

#### 3.1.6 Impact—How Were Decisions Made About Capturing and Evaluating the Impact Arising From the CIPs and What Were the Impacts?

Members of the HPRU public involvement team, presented a range of possible evaluation resources including the Public Involvement Impact Assessment Framework (PiiAF) [35], the Cube Evaluation Framework [36] and Public Involvement Impact Logs [37]. The Public Involvement Impact Log is a resource that has been developed by colleagues in the People in the Health West of England (People in the Health West of England [PHWE] is a collaboration led by the University of West of England. It is a network that brings together key research partners from across the NIHR and beyond to work jointly on public involvement) to capture reflections on public involvement practice and the range of possible outcomes and subsequent impacts of public involvement, including those on the research and for the individuals involved [37, 38]. The researchers agreed on using the impact logs as they provided a simple framework that allowed the impacts to be captured from the researcher's and community members’ perspectives. This approach was also considered useful as the information provided on the logs could relate to impacts on the projects as well as the learnings for the individuals. One of the researchers working on a CIP adapted the impact log and created a digital version of the log to include additional elements of the Public Involvement Impact Assessment Framework (PiiAF) [39]. This log was completed by the researcher after each workshop and Parent Advisory Group (PAG) session. The PAG group would use the log to review their journey towards achieving the anticipated outcomes at the start of each meeting. By doing so, this enabled the group to adjust their actions in a participatory and learning process.

The regular CIP meetings provided a space for continuous reflection throughout the process on the range of impacts and challenges.

## 4 Discussion

This paper outlines the processes that were undertaken to set up a CIP scheme. Existing guidance on community involvement and engagement does not often include detailed information about the steps necessary to set up an infrastructure for working with under‐served communities in public health research [16, 40]. Our work addresses this gap by detailing the necessary time, resources, and strategies required to develop meaningful relationships between researchers and community members.

A critical factor that has led to success of the CIPs has been the emphasis on investing time to establish relationships and build trust with the communities to understand how they would like to be involved. In the project working with farmers, this was facilitated by initially engaging with someone who was already a trusted member of that community and who could act as a gatekeeper in building further relationships. Our approaches, guided by the input of the communities and supported by an organisational infrastructure, have resulted in ongoing opportunities and collaborations on projects within and outside of the HPRU. It is our aspiration that these projects will pave the way for the development of new mechanisms to help bridge gaps between organisational research priorities and research priorities recognised as important by communities. However, this potential will only be fully realised in the longer term if research infrastructures at both a local and national level become more flexible and develop diverse channels through which to engage with different underserved communities.

The CIPs outlined in this paper have highlighted the important need for organisations to invest time and resources to support relationship‐building between researchers and community members. This further corroborates recommendations in current guidance and literature [20, 21, 41].

In the existing literature and guidance, whilst resources are mentioned as an important factor in promoting inclusion, they do not provide specific detail on the practicalities of what resources are required when working with under‐served communities [21, 22]. This level of detail could be helpful for informing researchers and organisations how much funding and time might be required when involving communities in research. Furthermore, the learnings from our projects reflect challenges that arose though the experience of undertaking community involvement.

For example, the nature of the scheme meant that some of the projects had limited timelines (see Table 1) and some projects received more funding than others. This presented a challenge as it was uncertain how the work with the communities would be sustained past the shorter project deadlines.

Community involvement, while invaluable, does come with risks. Instances of miscommunication or misunderstanding may arise when researchers are not upfront and transparent about what can be achieved and what is possible through the research and their involvement [42]. Furthermore, the lack of feedback or information sharing following involvement from community members may result in community members feeling used for a specific purpose, particularly if there are few benefits for that community from involvement. As a result, these actions could be positively damaging, leading to distrust and disengagement from the community and lack of willingness to be involved in future research projects [16].

The CIP team members are collaborating with colleagues across the NIHR and partner organisations to explore solutions to these challenges. For example, community members from project 4 have been invited to join the HPRU BSE Public Involvement Strategy Group. Individuals who were involved in project 2 have been invited to join the local Young People's Advisory Group which offers young people opportunities to be involved in supporting a range of research projects.

We would encourage others to be mindful of these considerations and more work needs to be done to develop opportunities that enable continued involvement beyond the end point of smaller projects. The points above reinforce the importance of transparency and developing reciprocal relationships which involve creating shared plans and manageable expectations, providing feedback, and offering short‐term paybacks [43, 44, 45].

Building relationships and trust with under‐served communities to foster research collaboration is crucial so that we can hear more of the voices of people we so often miss. This relationship‐building is critical, and too often it is not done well, and research continues to suffer from lack of representation as a result [10, 41]. The more time we take to build relationships and trust in communities, the more likely we are to engage the most under‐served/stigmatised groups in the future, though for some topics and/or groups this is likely a very long road.

The reflections from setting up this CIP scheme also echo the importance of flexibility when working with under‐served communities [41]. For example, in the paper by Gafari et al. flexibility was necessary for adapting their recruitment strategies, so they accommodated the needs and preferences of the communities [41]. Our work supports this recommendation and reflects the various recruitment approaches reported in this paper. Furthermore, through these projects, we have also acknowledged that if there are certain topics discussed that may be sensitive or controversial in nature there should be alternative mechanisms in place to ensure that individuals can contribute using their preferred method. That stated, even with alternative methods of involvement, there are likely to be topics/groups of people that we may not successfully reach, for example, some people may be concerned about vaccines and worried that they will be labelled as ‘anti‐vaxxers’.

Working with communities has also highlighted the important need for flexibility with regard to the strategies used to communicate with, and involve, communities. For example, using a WhatsApp group instead of email for communication and considering religious events such as Ramadan and Eid when planning meetings (as experienced by the researcher working on the MAVIS project). Online meetings have been advantageous for the inclusion of public contributors who needed to be recruited from sites across the UK (this was the case for project one ‘Hold the door open’) or who did not want to or could not attend face‐to‐face meetings easily. Furthermore, for those who may have been shielding, this approach has ensured they have not been disadvantaged or excluded.

However, the lack of in‐person meetings when initiating the community projects proved challenging when trying to reach out to community organisations, community leaders and certain communities. For example, the farming community who often work long and inflexible hours. Without existing relationships, it was difficult to make initial contact with external community‐based organisations. The researchers working on project four have only held face‐to‐face meetings as without these in person meetings, it would not have been possible to engage the group. Furthermore, by conducting meetings online, members without access to internet or the technology to join meetings online may have been excluded from participating, limiting their involvement in the projects. The researchers working on project 3 ‘Engaging the farming community in zoonotic disease research’ wanted to ensure that they could maximise outreach and engagement from the farming community despite their demanding schedules. To achieve this, they employed a stepwise approach that involved validating their ideas with the community members. Through this iterative process, they were able to progressively improve their approach and achieve greater success in engaging with the farming community.

When working on a project with limited timelines, flexibility can be challenging. However, knowledge of the communities and their commitments at the earliest phase may help with planning future community/public involvement activities to ensure they can be undertaken within project timescales.

The proposals for the CIP projects were developed by HPRU BSE researchers, meaning that the researchers determined what communities they worked with and how the projects were developed. Reflecting on the issues noted above, our future work will explore opportunities to provide funding for community organisations to prioritise projects that they consider important, which would be supported by HPRU researchers. This grassroots approach could lead to more sustainable ways of working with communities, build capacity and ensure research is truly reflective of the needs of our population.

In addition to adapting our current model, we will be collating the evaluations from the CIP projects and developing approaches to share our findings. We will work with our community members to use various dissemination strategies including publications, blogs, webinars and videos.

From this work, we have also developed key recommendations for others who are setting up their own infrastructures for working with under‐served communities in public health research (see Table 4).

**TABLE 4:** Key recommendations for developing an infrastructure for under‐served communities involvement in public health research.

Key Recommendations
1. Organisations and research funders should provide support, funding and resources that enable researchers to invest preparatory time, outside of specific projects, that is solely for the purpose of establishing trust and maintaining relationships with under‐served communities.
2. Researchers should be flexible and work collaboratively with under‐served communities to ensure that the planned approaches to involvement are responsive and accommodating of the needs and preferences of the community. This may involve consideration of both practical and cultural factors.
3. Training should be provided for both researchers and contributors from under‐served communities in areas related to the research project and public involvement. This creates opportunities for continuous learning, CV and skill development for the people we work with and promotes equity and reciprocal relationships between the researchers and communities. 4. Continuous evaluation of community involvement practices identifies areas for improvement, providing opportunities to share successes and challenges in real‐time. These evaluation exercises need to address issues that are relevant to the communities being engaged with, rather than academic debates about impact, provide timely feedback and not make onerous demands in terms of time or resources. 5. Developing relationships with key people who are trusted within an under‐served community and who can act as a mediator between researchers and a particular community, if possible, is a key early step. 6. Research projects, where possible, should address issues and problems that a particular under‐served community recognise as important and produce guidance and information that is relevant to tackling these problems in a timely fashion. 7. Reciprocity provides the social grammar that underpins successful relationship building with under‐served communities. What this looks like needs to be determined by the community being worked with. It may, for example, take the form of a user led piece of research but frequently will take the form of smaller and more immediate paybacks such as creating space within the project to support social activities or providing information and support to access healthcare.

## 5 Conclusion

This paper builds on existing work exploring how researchers can build meaningful and sustainable partnerships with under‐served communities to better understand key health issues and promote the inclusion of diverse voices in public health research.

In this paper, we have presented the first steps taken to involve and work with under‐served communities across four projects and make recommendations based on our learnings. To close the gap between the ‘system’ and our wider communities it is important to realise and appreciate the investment and necessary steps required from the earliest phase.

## Author Contributions

**Carmel McGrath:** Conceptualization; Writing–review & editing; Writing–original draft; Methodology; Project administration. **Gemma Lasseter:** Conceptualization; Writing–original draft; Writing–review & editing; Methodology; Project administration. **Noreen Hopewell‐ Kelly:** Conceptualization; Writing–original draft; Writing–review & editing; Methodology; Project administration. **Emma Anderson:** Conceptualization; Writing–original draft; Writing–review & editing; Methodology. **Ellen Brooks‐Pollock:** Conceptualization; Writing–original draft; Writing–review & editing; Methodology. **Hannah Christensen:** Conceptualization; Writing–original draft; Writing–review & editing; Methodology. **Sarah Denford:** Conceptualization; Writing–original draft; Writing–review & editing; Methodology. **Rosie Essery:** Conceptualization; Writing–original draft; Writing–review & editing; Methodology. **Shoba Dawson:** Conceptualization; Writing–original draft; Writing–review & editing; Methodology. **Evelyn Schiller:** Conceptualization; Writing–original draft; Writing–review & editing; Methodology. **Taru Silvonen:** Conceptualization; Writing–original draft; Writing–review & editing; Methodology. **Christina Stokes:** Conceptualization; Writing–original draft; Writing–review & editing; Methodology. **Amy Thomas:** Conceptualization; Writing–original draft; Writing–review & editing; Methodology. **Clare Thomas:** Conceptualization; Writing–original draft; Writing–review & editing; Methodology. **Andy Gibson:** Conceptualization; Writing–original draft; Writing–review & editing; Methodology; Project administration. All authors contributed to drafting and editing the article and approved the final manuscript.

## Ethics Statement

As per the Health Research Authority/NIHR INVOLVE statement, ethical approval was not required for this patient and public involvement piece. NHS Health Research Authority. What do I need to do? [Online]: Health Research Authority; 2020 [updated December 2020; cited 2023 September]. Available from: https://www.hra.nhs.uk/planning-and-improving-research/best-practice/public-involvement/what-do-i-need-do/ [46].

## Conflicts of Interest

The authors declare no conflicts of interest.

## Data Availability

All data generated or analysed during this work are included in this published article.
